# Evaluation of the relationship between NIRS (near-infrared spectroscopy) and optic nerve sheath diameter measurement in children with increased intracranial pressure: a pilot study

**DOI:** 10.1186/s13052-021-01035-2

**Published:** 2021-04-10

**Authors:** Didar Arslan, Dinçer Yıldızdaş, Özden Özgür Horoz, Nagehan Aslan, Faruk İncecik

**Affiliations:** 1 Department Pediatric Intensive Care, Cukurova University Faculty of Medicine, Adana, Turkey; 2 Department of Pediatric Neurology, Cukurova University Faculty of Medicine, Adana, Turkey

**Keywords:** Children, Increased intracranial pressure, Near infrared spectroscopy, Optic nerve sheath diameter

## Abstract

**Background:**

The increased intracranial pressure (ICP) syndrome may emerge depending on many different neurological factors and the early diagnosis and treatment are important for the prevention of neurologic damage and related mortality. In recent years, the follow-up of increased ICP with non-invasive methods has been rising. In this study, our objective was to determine the significance and any possible correlation between Optic Nerve Sheath Diameter (ONSD) and Near Infrared Spectroscopy (NIRS) in children with increased ICP.

**Methods:**

Patients who were hospitalized in our pediatric ICU at Çukurova University Medical Faculty between June 2018 and June 2019 due to the suspicion of increased ICP were included in this study. The demographic characteristics of patients, diagnosis at admission, results of the cranial CT and MRI examinations, and results of the simultaneous ONSD and NIRS measurements were recorded.

**Results:**

A total of 36 patients were included in our study. With respect to the diagnosis, non-traumatic causes were at the forefront in 30 patients (83.3%), and the most common causes were meningoencephalitis (*n* = 9; 25%) and non-traumatic bleeding (*n* = 7; 19.4%). Six of the patients were under the age of one year (16.7%), and the mean values of ONSD and NIRS were 4.8 ± 0.7 mm and 71.1 ± 12.4% respectively in this group. Fourteen patients were in the one to ten year age group and the mean values of ONSD and NIRS were 6.1 ± 0.6 mm and 72.7 ± 9.3% respectively. Sixteen patients were over ten years of age (44.4%), and the mean values of ONSD and NIRS were 5.6 ± 0.7 mm and 74.2 ± 16% respectively. There was no correlation between the ONSD and NIRS values (r:0.307; *p* = 0.068).

**Conclusion:**

Our study showed that ONSD measurements were helpful in children with increased ICP and reflected the increase in ICP. However, our study also demonstrated that ONSD was not in correlation with the NIRS monitoring. We believe that there is a need for further studies focused on the use of ONSD and NIRS in the monitoring of increased ICP.

## Introduction

Increased intracranial pressure (ICP) syndrome may emerge depending on many different neurological factors, e.g. trauma, infection, hydrocephaly, toxic encephalitis, brain tumor, vasculitis or idiopathic. Early diagnosis and treatment are important for the prevention of neurologic damage and related mortality. In ICP, the measurement of intraparenchymal and intraventricular pressure with a special catheter is the gold standard for the determination of intracranial pressure. However, it is rather uncommon in clinical practice due to complications such as invasive process and infection [1, 2]. Consequently, there is a need for non-invasive methods, which can be used in the monitoring of ICP, especially in pediatric patients. Several different non-invasive monitoring methods—such as computerized tomography (CT), magnetic resonance imaging (MRI), transcranial doppler ultrasonography, near-infrared spectroscopy (NIRS), and ultrasonographic optic nerve sheath diameter (ONSD) measurement—are currently available [3].

The popularity of ONSD measurement has increased in recent years due to its non-invasive process and repeatability. The optic nerve is anatomically part of the central nervous system. It is surrounded by dura mater, subarachnoid space, and cerebrospinal fluid. Therefore, any change in the ICP also changes the diameter of the optic nerve sheath [4–6].. In a study focused on the eighteen adult patients with traumatic brain injury, all patients had invasive ICP monitoring and they determined that the diameter of the optic nerve sheath increased simultaneously with an increased ICP and decreased back to the baseline diameter in the same rate as the ICP [7]. High ICP can occur in the absence of papilledema and appearing time of the papilledema is different depending on the reason. Papilledema that develops in patients after head trauma may develop immediately, occur several days after the injury, or up to 2 weeks later [8]. In patients with increased ICP, the diameter of the optic nerve sheath increases before the development of the papillary edema [9].

The near-infrared spectroscopy is another non-invasive method, which provides information about the regional changes in tissue oxygenation [10]. In a study, investigators compared the changes in NIRS with the invasive pressure measurement method and showed that NIRS changed parallel to the increase in ICP [11].

Here we aimed to determine the significance of ONSD and NIRS in children with increased ICP and any possible correlation between these non-invasive methods in our tertiary pediatric intensive care unit (PICU).

## Methods

Patients who were hospitalized in our PICU at Çukurova University Medical Faculty between June 2018 and June 2019 due to the suspicion of increased ICP were included in this study. We included patients with traumatic brain injury, suspected meningoencephalitis, non traumatic intacranial bleeding, clinical signs of suspected increased intracranial pressure. The study was performed prospectively. Increased ICP was suspected in the enrolled patients due to the clinical findings (change in consciousness level, dilated pupils unresponsive to light, loss of the brainstem reflex, cranial nerve injury, and Cushing’s triad) or radiological findings (shift, effacement in sulci, ventricular collapse, and compression in cisterns). The demographic characteristics of patients, diagnosis at admission, results of the cranial CT and MRI examinations, and results of the simultaneous ONSD and NIRS measurements were recorded. Children with ocular trauma and optic nerve disease were excluded from the study. Parents of children were informed during the intensive care hospitalization and written consent was obtained.

**Fig. 1:**
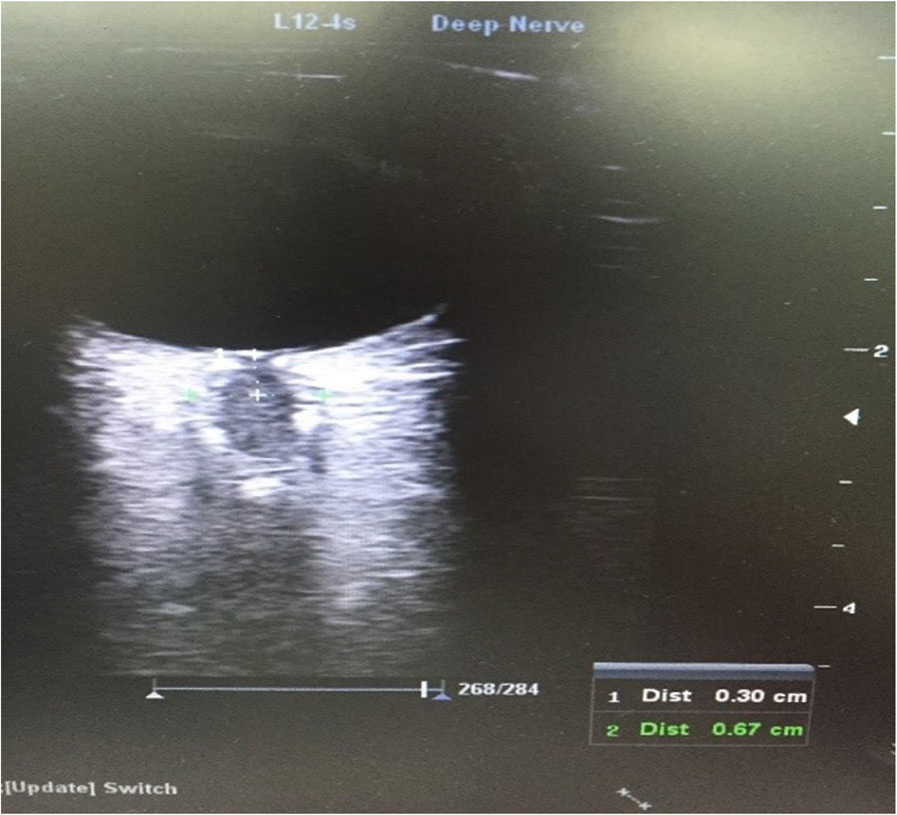
Ultrasonographic ONSD measurement

**Fig. 2:**
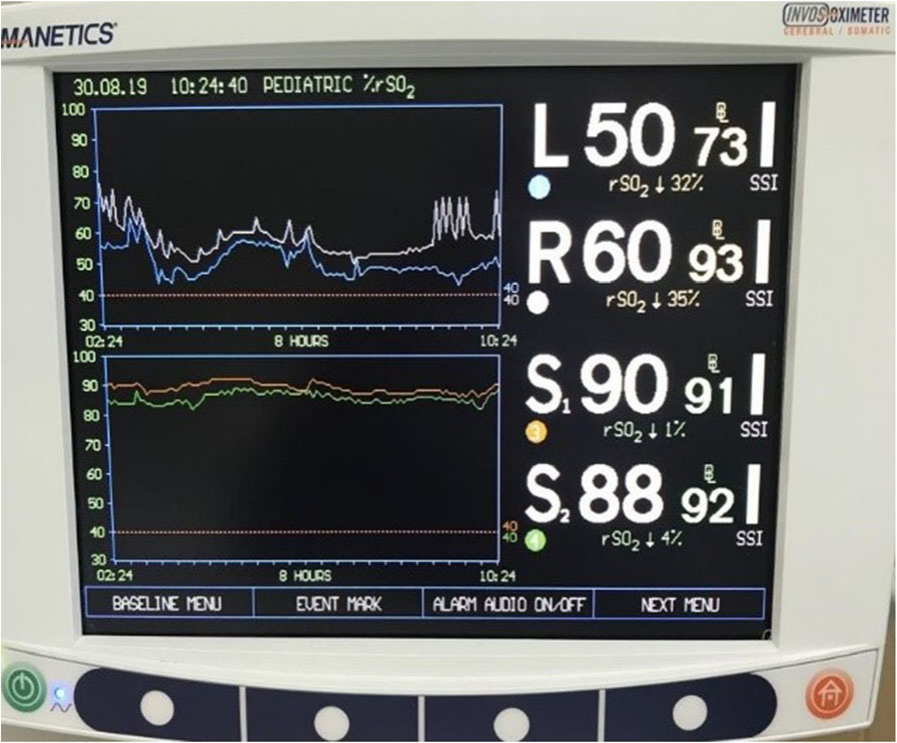
Cerebral NIRS measurement

A 7 MHz linear probe and Mindray® Ultrasound device were used in this study. The measurements were taken by two PICU fellow experienced in the optic nerve ultrasonography, and the mean value of the measurements were calculated. During the measurements, the bedhead was adjusted to zero degrees, while the head of the patient was positioned at the midline. The measurements were performed in both eyes at regions 3 mm below the bulbs, while the eyes were closed, and the mean value of these measurements was calculated (Fig. 1). The INVOS® 5100C Cerebral Oximeter (Somanetics, Troy, MI) was used for the NIRS, which demonstrates the regional oxygenation level of the cerebral tissue (Fig. 2). NIRS measurements were made by a disposable sensor that placed on frontal area of head.

The study was approved by the Ethics Committee for Clinical Research in Cukurova University Medical Faculty (Date: 13.01.2017; No: 60).

### Statistical analysis

The statistical analysis was carried out via the SPSS v23.0 software package. The intergroup comparisons of the categorical variables were performed with Chi-square tests. The intergroup comparison of the variables with non-normal distribution was done with a Mann-Whitney U test, while correlation between the measurements was assessed with Spearmen’s Correlation Coefficient. The accepted limit of significance was *p* < 0.05.

## Results

A total of 36 patients were included in our study. The mean age was 99.5 ± 72.8 months (1–201 months). Fifteen (41.7%) patients were females. With respect to the diagnosis, non-traumatic causes were at the forefront in 30 patients (83.3%), and the most common causes were meningoencephalitis (*n* = 9; 25%) and non-traumatic bleeding (*n* = 7; 19.4%). ICP increase after head trauma was detected in six patients (16.7%) and they had no ocular trauma. Less common diagnoses were postarrest, intracranial mass lesion, and infarction of incidence. A total of 25 patients (69.4%) were intubated, and the mean GCS score was 7.1 ± 2.99. Thirty-one patients (86.1%) underwent cranial CT and 25 (69.4%) underwent cranial MRI. Cranial CT displayed increased ICP findings in 25 patients (69.4%) and cranial MRI revealed findings in 22 patients (61.1%). The demographic characteristics and other findings are listed in Table 1. Ten patients died (27.8%). The mean duration of hospitalization was 18.3 ± 17.8 days. ONSD and NIRS measurements according to age groups of patients are listed in Table 2. We did not find any correlation between sex and mortality rate. We did not find any correlation between mortality and method of diagnosis (cranial CT or MRI). There was no significant correlation between ONSD and NIRS values (r:0.307; *p* = 0.068). We did not find any significant correlation between ONSD or NIRS and the duration of hospitalization. (r = − 0.291 *p* = 0.085, and r = − 0.146 *p* = 0.396). We did not find any correlation between ONSD or NIRS and mortality rate.

**Table 1:** The demographic characteristics and findings of patients

	N (%)
Age (years)	
≤ 1	6 (16,7)
1–10	14 (38,9)
≥ 10	16 (44,4)
Gender	
Female	15 (41,7)
Male	21 (58,3)
Diagnosis	
Trauma	6 (16,7)
Non-trauma	30 (83,3)
Meningoencephalitis	9 (25)
Intracranial bleeding	7 (19,4)
Postarrest	4 (11,1)
Intracranial mass	3 (8,3)
Others	7 (19,4)
Cranial CT	31 (86,1)
+^a^	25 (69,4)
-^b^	6 (16,7)
Cranial MRI	25 (69,4)
+^a^	22 (61,1)
-^b^	3 (8,3)
Mortality	10 (27,8)

^a^ the number of patients with signs of increased intracranial pressure

^b^the number of patients without signs of increased intracranial pressure

**Table 2:** ONSD and NIRS measurements according to the age groups of patients

Age (years)	N(%)	ONSD (mm)	NIRS (%)
≤1	6 (16,7)	4,8 ± 0,7	71,1 ± 12,4
1–10	14 (38,9)	6,1 ± 0,6	72,7 ± 9,3
≥10	16 (44,4)	5,6 ± 0,7	74,2 ± 16

## Discussion

Increased intracranial pressure syndrome may emerge due to many different neurological factors. Clinical findings like changes in consciousness level, dilated pupils unresponsive to light, loss of the brainstem reflex, cranial nerve injury, Cushing’s triad, or radiological findings such as shift, effacement in sulci, ventricular collapse, and compression in cisterns may be encountered. Early diagnosis and treatment are important for the prevention of neurologic damage and related mortality. In ICP, the measurement of the intraparenchymal, intraventricular pressures with a special catheter is the gold standard for the determination of the intracranial pressure. However, it is rather uncommon in clinical practice due to complications such as invasive process and infection [1, 2]. Therefore, there is a need for non-invasive methods, which may be used in the monitoring of ICP in pediatric patients.

Ultrasonographic ONSD measurements became increasingly popular as a result of its early detection of increased ICP, non-invasive process, repeatability, and implementation at bedside. There are many studies in the literature focused on the ONSD measurements in patients with increased ICP. Although there are some conflicting results, all studies demonstrated that ONSD increased in patients with increased ICP. Ballantyne et al. [12] conducted a study of 102 healthy children and found that ONSD was between 2.1–4.0 mm in children under the age of one year and between 2.1–4.3 mm in children over the age of one year. They reported cut-off values of > 4.0 mm and > 4.5 mm in children under and over the age of one year respectively. Malayeri et al. [13] conducted a study in 78 ill and 78 healthy children; dividing them into two age groups (<four years and > four years), they found that ONSD was 5.5 ± 0.6 mm (<four years) and 5.6 ± 0.7 mm (>four years) in children with the increased ICP, while the same values were 3 ± 0.6 mm (<four years) and 3.6 ± 0.4 mm (>four years) in healthy children. Beare et al. [14] conducted a study of 21 African children with neurological disease and found that ONSD was 4.3–6.2 mm in children with the clinical and/or cranial CT findings of the increased ICP, while the same value was 2.8–4.4 mm in children with neurological disease and negative clinical and cranial CT results. They reported 4.2 mm as the upper limit for ONSD and stated that ≥4.5 mm should be accepted as the cut-off value for increased ICP. Padayachy et al. [15] conducted a study of 174 pediatric patients and evaluated invasive ICP and ONSD. They found that in patients with ICP > 20 mmHg, ONSD was 5.6 mm, 5.92 mm and 5.75 mm in the age groups <one year, one to four years and > four years respectively. Rehman Siddiqui et al. [16] conducted a study of 48 children with increased ICP and determined ONSD values of 4.64 ± 0.48 mm (cut-off> 4 mm) for the age group under one year; 6.44 ± 0.65 mm (cut-off> 4.71 mm) for the one to ten year age group and 6.28 ± 0.62 mm (cut-off> 5.43 mm) for the age group over ten years. In a study conducted in 2019, they evaluated ONSD measurements in children with acute liver failure. 41 children with acute liver failure and 47 healthy children were taken. Those with acute liver failure are also grouped as with and without hepatic encephalopathy (HE). ONSD was 4.2 mm, 4.4 mm and 5.2 mm in controls, ALF without HE and with HE. In children with acute hepatic failure, the presence of ONSD above 5.1 mm is considered significant for hepatic encephalopathy [17]. In a study conducted in 2020, ONSD and ICP measurements were examined in 72 children who underwent neurosurgery operation. They determined that the best ONSD cut-off value for detecting ICP ≥ 15 and ≥ 20 mmHg was 5.28 and 5.57 mm [18].

However, in the first studies focused on this topic, it was stated that these values were low for increased ICP and higher ONSD values should be considered in children with increased ICP. In our study, six patients were under the age of one year (16.7%), and their mean ONSD value was 4.8 ± 0.7 mm. The mean ONSD values were 6.1 ± 0.6 mm and 5.6 ± 0.7 mm in 14 children (38.9%) in the age group one to ten years and in 16 children (44.4%) in the age group >ten years respectively (Table 2). As in the previous studies, the ONSD values have been increased.

In a study focused on the eight adult patients with cranial trauma and brain injury, the authors determined a significant difference between patients with ICP > 25 mmHg (67 ± 1%) and ICP < 25 mmHg (71 ± 2%) for NIRS values and following the hyperoxia test, a significant increase in NIRS values was determined in the low-ICP group, while no significant increase in NIRS values was noticed in the high-ICP group [19]. Adelson et al. [20] conducted a study of ten children with serious cranial trauma and reported that an increase in ICP led to an increase in cerebral blood flow, cerebral vasodilation, and oxyhemoglobin. In addition, they warned that the opposite might occur in patients with very high ICP levels. Lewis et al. [21] conducted a study in ten adult patients with severe cranial trauma and evaluated whether NIRS decreased parallel to the decrease in the jugular bulb venous oxygen saturation (< 55%), and they did not find any significant decrease in NIRS values. In a study conducted in 31 adult patients with serious cranial injury, the changes in NIRS, intracranial pressure (ICP) and cerebral perfusion pressure (CPP) were compared after hyperoxia, hypocapnia, and mannitol administration, and it was found that the changes in NIRS were not significant as in other parameters except for hyperoxia [22]. Zuluaga et al. [11] conducted a study in 30 children, who had increased ICP and underwent invasive monitoring (ICP), and found that NIRS value was 75.2 ± 10.1% and 70.8 ± 12.5% in patients with ICP < 20 mmHg and ICP > 20 mmHg respectively. They reported that NIRS decreased along with the increase in ICP, but there was no significant correlation between these parameters (*p* = 0.3). Likewise, we did not find a significant correlation between increased ICP and NIRS in our study.

In a PuBMed based literature search showed us there are just limited number of studies in the literature related to the usage of NIRS in patients with increased ICP, and they reported conflicting results. There is no published study focusing on the concomitant evaluation of both ONSD and NIRS. Our study is the first pediatric study focused on the ultrasonographic ONSD measurements and NIRS values in patients with increased ICP.

The main limitation of our study is the limited number of patients and ONSD values are not yet standardized. Another limitation of our study is that failure to routinely apply invasive ICP monitoring in our PICU due to the lack of an experienced neurosurgery team in our hospital. Therefore, the correlation between the changes seen in ICP with invasive ICP monitoring and the ONSD and NIRS could not be done.

## Conclusion

In conclusion our study showed that ONSD measurements were helpful in children with increased ICP and reflected the increase in ICP. However, our study also demonstrated that ONSD was not in correlation with the NIRS monitoring. We believe that there is a need for further studies focused on the use of ONSD and NIRS in the monitoring of increased ICP.

## Data Availability

Please contact author for data requests.
